# Polarization-maintaining reflection-mode THz time-domain spectroscopy of a polyimide based ultra-thin narrow-band metamaterial absorber

**DOI:** 10.1038/s41598-018-20429-7

**Published:** 2018-01-31

**Authors:** Maria Denise Astorino, Renato Fastampa, Fabrizio Frezza, Luca Maiolo, Marco Marrani, Mauro Missori, Marco Muzi, Nicola Tedeschi, Andrea Veroli

**Affiliations:** 1 Department of Information Engineering, Electronics and Telecommunications, “ La Sapienza” University of Rome, Via Eudossiana 18, 00184 Roma, Italy; 2 Dipartimento di Fisica, “ La Sapienza” University of Rome, Piazzale Aldo Moro 5, 00185 Roma, Italy; 3 Istituto dei Sistemi Complessi, Consiglio Nazionale delle Ricerche, Unità Sapienza, Piazzale Aldo Moro 5, 00185 Roma, Italy; 4 Istituto per la Microelettronica e Microsistemi - Consiglio Nazionale delle Ricerche, Via Fosso del Cavaliere 100, 00133 Roma, Italy; 5 Engineering Departmental Faculty, Campus Bio-Medico University of Rome, Via Alvaro del Portillo 21, 00128 Roma, Italy; 6 Centro Fermi - Museo Storico della Fisica e Centro Studi e Ricerche “ Enrico Fermi”, Piazza del Viminale 1, 00184 Roma, Italy

## Abstract

This paper reports the design, the microfabrication and the experimental characterization of an ultra-thin narrow-band metamaterial absorber at terahertz frequencies. The metamaterial device is composed of a highly flexible polyimide spacer included between a top electric ring resonator with a four-fold rotational symmetry and a bottom ground plane that avoids misalignment problems. Its performance has been experimentally demonstrated by a custom polarization-maintaining reflection-mode terahertz time-domain spectroscopy system properly designed in order to reach a collimated configuration of the terahertz beam. The dependence of the spectral characteristics of this metamaterial absorber has been evaluated on the azimuthal angle under oblique incidence. The obtained absorbance levels are comprised between 67% and 74% at 1.092 THz and the polarization insensitivity has been verified in transverse electric polarization. This offers potential prospects in terahertz imaging, in terahertz stealth technology, in substance identification, and in non-planar applications. The proposed compact experimental set-up can be applied to investigate arbitrary polarization-sensitive terahertz devices under oblique incidence, allowing for a wide reproducibility of the measurements.

## Introduction

Terahertz (THz) technology has recently received growing attention, enabling it to overcome the so-called “ THz gap” thus connecting the fields of electronics and optics^1^. While the microwaves and the far-infrared frequency regions immediately below and above the THz band, respectively, have been extensively investigated, the THz frequencies are still the aim of the research breakthroughs. An intense interaction between the disciplines of physics and engineering has made it possible to generate and detect broadband coherent THz radiation. In particular, THz time-domain spectroscopy (THz-TDS) has matured into an extremely useful tool for characterizing THz devices, thanks to its non-destructive and non-invasive features^2–4^. The non-ionizing nature of the THz frequencies has promoted advanced applications in medical diagnoses and in material science, allowing it to identify different substances through their specific absorption patterns^5,6^. THz devices have also been successfully applied in security detection^7–9^, because non-conductive and non-polar materials, such as packaging, plastics, paper and ordinary clothes, show low absorption at these frequencies.

In this context, the THz regime represents a fertile area for the development of metamaterial absorbers (MMAs)^10–13^, which are manmade devices designed to absorb specific bands of the incident electromagnetic radiation and generally constituted of periodic structures with sub-wavelength unit cells^14^. Their customizable optical characteristics^15–17^ are not observed in their constituent materials, but they can be artificially manipulated by employing electromagnetic resonators. This resonant spectral feature is of particular interest at THz frequencies, where it is difficult to find natural materials with both narrow absorption bands and high absorption coefficients^18^. In addition, since the unit cell sizes necessary for the THz frequencies are of the order of tens of micrometers, they can be reproduced with high precision through micro-fabrication processes based on standard photolithographic techniques. This can be very useful in the development of various MMAs and applications, such as highly sensitive biochemical sensors^19^, microbolometers of thermal detectors^20,21^, THz stealth technology^22^, and THz imaging^23^.

By exploiting the principle of scalability of these devices, once proved their feasibility, it is conceivable to extend their use even at higher frequencies where the tolerances required in manufacturing processes are more critical. A specific-designed MMA could be also dimensioned to operate over a wide range of frequencies spanning from microwave, THz, to the infrared (IR), and optical spectrum^24,25^.

In order to assess a benchmark for future MMAs development, it is fundamental to investigate the underlying physics on simple three-layer metal-dielectric-metal devices in which the interplay between the metallizations through the dielectric layer can be more easily studied as a function of incidence angle and polarization. In general, it is essential to maximize the losses and consequently the absorbance *A*(*ω*), where *ω* is the angular frequency, in narrow spectral bands. According to the relation *A*(*ω*) = 1 − *R*(*ω*) − *T*(*ω*)^26^, this can be obtained through the simultaneous minimization of the reflectance *R*(*ω*) by matching the impedance of the MMA to that of free space at a desired frequency, and of the transmittance *T*(*ω*) by employing a metallic ground plane. This achievement normally requires first a preliminary study in which the characteristics of the resonator are defined in connection with the material properties of the middle spacer; then followed by an optimization phase in which the geometrical features of the resonator and the thickness of the dielectric layer are adjusted for the best performance. Following the design phase, an accurate experimental characterization of MMA electromagnetic response is needed. This makes the THz spectroscopy fundamental both to demonstrate the functionality of the MMAs and to study their compliance with the design features.

With the aim of implementing a valid THz experimental set-up for an MMA as described above, it is necessary to solve some significant problems. Due to the presence of a lower metallic ground plate which prevents transmission of THz radiation through the device, measurements should be conducted in reflection-mode. This configuration needs a higher number of THz optical components as compared to the more easily configurable transmission-mode set-up^4^. The quality of the experimental results critically depends on alignment errors of THz optical components which might alter the measured signals due to differences in THz pulses path length and phase errors.

In this paper, we show how it is possible to realize and characterize at THz frequencies an ultra-thin ground-plane-backed MMA^27^ with an extremely selective absorbance spectrum. A specifically designed polarization-maintaining reflection-mode THz-TDS set-up with innovative features is employed for its electromagnetic characterization. This three-layer metal-dielectric-metal configuration has been chosen for the MMA to simplify the fabrication processes and to avoid misalignment problems with respect to other more complex multi-layer configurations already published^23,26^. This approach reduces the number of experimental degrees of freedom in the fabrication process, allowing a more straightforward controllability of the prototype stage. The choice of polyimide, as the dielectric spacer, also takes into account its possible application as a flexible MMA, adaptable to non-planar surfaces. The unit cell of the proposed MMA has been designed to exhibit a single resonance in the THz frequency range accessible to the custom reflection-mode THz-TDS set-up, providing a well-recognizable experimental absorption signal particularly useful for comparison with numerical simulations. Furthermore, the wide reproducibility found in the measurements proves our THz-TDS set-up to be potentially effective to accurately characterize arbitrary THz polarization-sensitive devices in reflection mode under oblique incidence. In fact, in the proposed reflection-mode THz-TDS system, only two identical plano-convex lenses and a custom aluminum prism reflector have been properly employed, thus avoiding the use of a complex set of parabolic mirrors to manipulate the THz optical path^2,28,29^, potential source of disturbance for both polarization and propagation of THz radiation.

## Results

### MMA description

The ultra-thin narrow-band MMA^27^ is realized with a three-layer metal-dielectric-metal configuration consisting of a patterned frequency-selective surface (FSS)^27,30^ inspired by ref.^23^, a highly flexible insulating polyimide spacer, and a metal ground plane on a silicon substrate. Polyimide PI-2611 from HD MicroSystems^31^ with nominal dielectric constant *ε′* = 2.9, loss tangent *tan δ* = 0.002, and thickness of 5.4 *μ*m, has been chosen as middle dielectric layer for its high electrical and thermal stability, low refractive index, low absorption, and flexibility^32–40^. It is a well-established material within the micro-fabrication processes, and it is used in applications of electronics on plastic and in photonic devices^41,42^.

**Figure 1:**
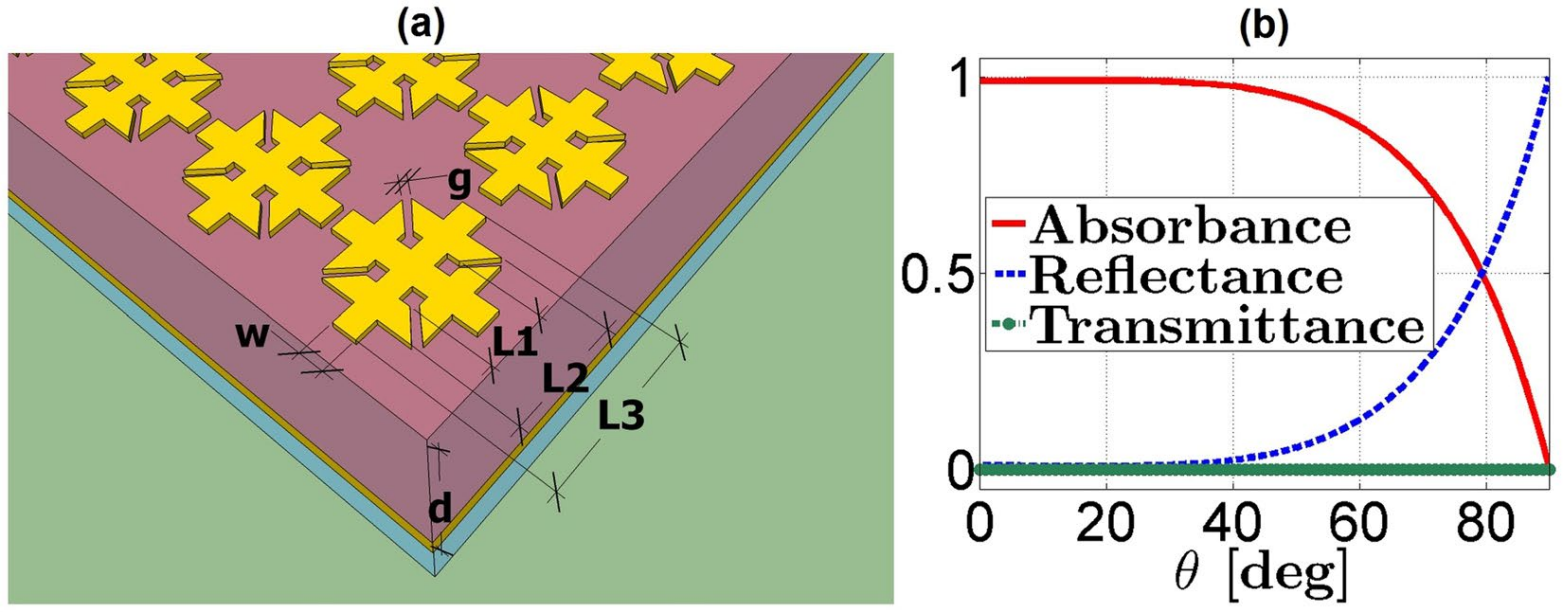
Schematic illustration of the ultra-thin narrow-band MMA and angular response in TE polarization. (**a**) Geometry of the narrow-band MMA with dimensions (in micrometer) *g* = 7, *w* = 11, *L*1 = 26, *L*2 = 50, *L*3 = 72, and *d* = 5.4. (**b**) Response as a function of the incidence angle *θ* at 1.09 THz in TE polarization with azimuthal angle *φ* = 0°.

The upper electric ring resonator (ERR)^23,27,30^ (with optimized dimensions specified in Fig. 1(a)) and the lower ground plane with 100 nm thickness are both made of lossy gold in order to ensure chemical stability and a high electrical conductivity (*σ* = 4.09 × 10^7^ S/m).

This ultra-thin MMA with unit cell of 80 *μ*m periodicity presents a significant subwavelength thickness of about *λ*_0_/49 at the absorbance frequency, where *λ*_0_ is the free-space wavelength.

The materials choice in the metamaterial design is a key point for the energy dissipation: in order to reach the desired absorbance, one needs to select highly conductive metals and highly insulating dielectrics, i.e., materials with low losses in the working frequency band. By adopting these material characteristics and the optimized dimensions, we have reached in the simulations a near unity absorbance at 1.09 THz, supposing a plane wave impinging on the unit cell under 16° oblique incidence in transverse electric (TE) polarization with the electric field parallel to the ERR arm (*φ* = 0°). These specific operating conditions have been implemented in the numerical simulations to take into account the restrictions imposed by the experimental set-up. However, it has been shown in ref.^27^ how the proposed narrow-band MMA exhibits a wide angular response for both TE and transverse magnetic (TM) polarizations as the incidence angle *θ* varies (see Fig. 1(b)), even if the TE case exhibits the more critical working conditions. Indeed, under oblique incidence in TE polarization, the absorbance levels undergo a monotonic decrease with the increase of the incidence angle, while in TM polarization the resonance peaks maintain high absorbance levels even under 80° incidence.

### Experimental set-up for polarization-maintaining reflection-mode THz-TDS

**Figure 2:**
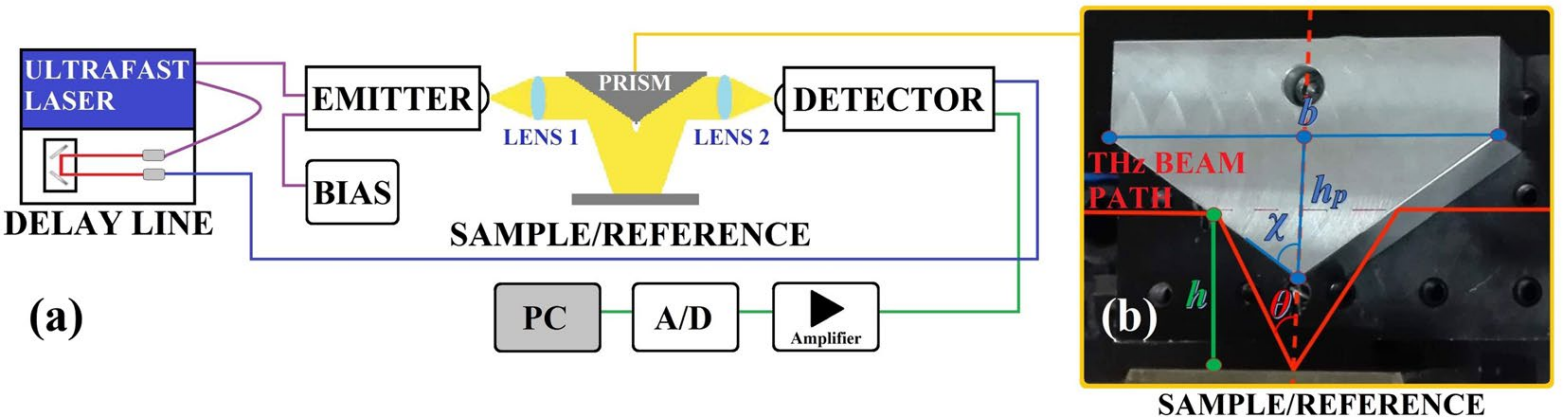
Sketch of the polarization-maintaining reflection-mode THz-TDS system and detail of the fabricated Al prism. (**a**) Experimental THz-TDS set-up and (**b**) geometry of the aluminum prism with *χ* = 54.7°, *h*_*p*_ = 19 mm, *h* = 38 mm, and *b* = 54 mm.

In order to characterize the electromagnetic behavior of the MMA in the THz range, we devised a THz-TDS system in reflection mode (see Fig. 2(a)). This set-up is based on a standard Menlo Systems (Germany) TERA K15 configuration^43–45^ and it is designed to minimize the mobile optical and mechanical parts (mirrors, lenses, and mechanical mountings). This is to avoid alignment errors, which might result in differences in THz pulses path length and phase errors, altering the measured signals and making difficult the comparison with the theoretical predictions. Terahertz radiation is generated by photoconductive antennae that allow the optical to THz signal conversion^4^. The antennae are excited by a femtosecond fiber-coupled laser (Menlo Systems T-Light) with an emission wavelength of 1560 nm, a repetition frequency 100 MHz, and pulse duration of nearly 90 fs. The remote control station of the THz-TDS system is constituted by an amplifier with the aim of amplifying the detected amplitude signal, an A/D converter and a PC for control and preliminary data processing (Fig. 2(a)).

For the purpose of realizing the reflection configuration, the THz beam line of the system was designed by using a couple of HRFZ-Si plano-convex lenses with effective focal length *f* = 25 mm and a custom metallic aluminum prism (Fig. 2(a)). The diverging THz radiation emitted by the emitter antenna is collected and collimated in a beam with a diameter of about 12 mm by means of the first lens (Lens 1 in Fig. 2(a)). Radiation collimated by Lens 1 impinges onto the prism and it is then reflected onto the sample (see Fig. 2(b)). Radiation reflected by the sample is reflected by the prism along the THz beam axis, while the second identical lens (Lens 2) permits the THz beam to be focused on the detector antenna. This realizes a reflection-mode collimated configuration in which the 12 mm diameter collimated beam of TE polarized THz wave impinges obliquely on the sample/reference with an angle *θ* of about 16° imposed by the prism geometry.

It is to be noted that the THz beam line of the reflection-mode THz-TDS system has been optimized employing only two identical plano-convex lenses and a custom aluminum prism reflector which determines the incidence and reflection angles on the MMA, thus maintaining the project requirements for a linearly polarized incident plane wave. Further, the choice of a compact set-up in which the THz radiation interacts with a small number of components (two lenses and a metal prism) is functional to minimize disturbing effects for both the polarization and the propagation of THz beam, which are instead likely present when one or more parabolic mirrors are used to manipulate the THz optical path.

The aluminum prism shown in Fig. 2(b), has been properly designed to allow the collimation of the THz beam on the sample following the derived relations:

1 $$\chi ={\cot }^{-1}\sqrt{1-\frac{{h}_{p}}{h}}$$

2 $$b=\frac{{h}_{p}}{\sqrt{1-\frac{{h}_{p}}{h}}}$$

where *χ* is the semi-angle of the lower vertex of the prism, *h*_*p*_ is its height, *h* is the distance between the axis of the incident THz beam on the prism and the sample surface, and *b* is the basis of the prism.

All the optical elements in the THz beam path were embedded in a dry nitrogen purged chamber at a relative humidity of about 4% in order to reduce the water vapor contribution, particularly present in the frequency band of interest. All spectroscopic measurements were performed at room temperature. The MMA (sample) and a reference made of an aluminum sheet of 970 *μ*m thickness were placed at about 36.5 mm from the lower vertex of the prism.

The electric field of the THz pulses propagating with the MMA sample and with the aluminum reference was measured. The THz pulses were averaged over 400 scans of the delay line with an overall acquisition time of 50 s and a delay line scan range of 100 ps. By applying the fast Fourier transform (FFT) to the acquired signals, the sample and reference THz electric field spectral amplitudes and phases were obtained as a function of frequency. The acquisition parameters of the THz set-up determines the frequency spectrum resolution Δ*f* = 1/(*N*Δ*t*) = 10.02 GHz, being the time resolution of the THz pulses Δ*t* = 33.36 fs and the measured data points *N* = 2992.

In the measurement set-up, the correct placement of the surfaces of the MMA sample and of the aluminum reference at the same distance from the lower vertex of the aluminum prism is fundamental in order to avoid differences in THz pulses path length and phase error. For this reason, a specific sample holder allowing longitudinal and perpendicular micrometric displacement with respect to the lower vertex of the aluminum prism was employed. Finally, in order to obtain the complex spectral reflection *S*_11_, i.e., the transfer function of the system which reports on the amplitude and phase changes due to absorbance and refraction of the MMA for a far-field characterization, we have divided the sample spectrum *E*_*sam*_ by the reference spectrum *E*_*ref*_:

3 $${S}_{11}=|{S}_{11}|{e}^{j\angle {S}_{11}}=\frac{|{E}_{sam}|}{|{E}_{ref}|}{e}^{j(\angle {E}_{sam}-\angle {E}_{ref})}.$$

The absorbance can be, therefore, obtained by: *A*(*ω*) = 1 − |*S*_11_(*ω*)|^2^ = 1 − *R*(*ω*), where reflectance is defined as *R*(*ω*) = |*S*_11_(*ω*)|^2^. This because the THz waves cannot be transmitted through the Si substrate due to the lower gold ground plane thicker than the penetration depth and, consequently, the transmittance of the MMA *T*(*ω*) = |*S*_21_(*ω*)|^2^ is negligible.

### Simulation and experimental results

One of the main aims of this work was to experimentally characterize the MMA in view of the optimizations of its performances under its more critical operating conditions, i.e., under oblique incidence in TE polarization^27^. In this case, the absorbance levels of the investigated MMA are typically lower, because the four-fold rotational symmetry^46,47^ of the resonator about the propagation axis cannot be fully exploited. Due to the presence of the lower metallic ground plate which prevents transmittance of the THz wave, we properly devised a compact polarization-maintaining THz-TDS set-up in reflection-mode able to guarantee a collimated configuration of the THz beam for oblique incidence analysis in TE polarization as described in the previous section.

**Figure 3:**
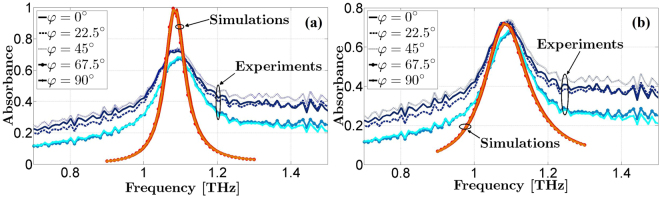
Comparison between simulated and experimental absorbance spectra as a function of the azimuthal angle *φ* with different polyimide loss tangent values under 16° oblique incidence in TE polarization. The simulations have been performed with the complex permittivity value (**a**) *ε*_*r*_ = 2.9 − 0.0058 *j* and with the estimated complex permittivity value (**b**) *ε*_*r*_ = 2.9–0.25 *j* for the polyimide layer.

Therefore the electromagnetic response of the ultra-thin MMA has been measured as a function of the azimuthal angle *φ* to verify the polarization insensitivity, rotating the sample of 0, 22.5, 45, 67.5, and 90 degrees with respect to the horizontal arm of the crossed-shaped ERR. As expected, thanks to the four-fold rotational symmetry of the MMA, the absorbance levels for the different values of *φ* are nearly equal and comprised between 67% and 74%, even under oblique incidence, as shown in Fig. 3(a) (see Supplementary Figs S1–S6).

The measured absorbance *A*(*ω*) differs from the near-unity value of the one simulated, and this discrepancy is mainly due to the values of the material parameters used in the simulation for describing the MMA. A possible reason for the mismatch between the simulations and the measurements can be attributed to higher Ohmic losses of the metal layers, being the electrical conductivity of thin Au films at THz frequencies much smaller than its bulk dc value^13,48^. Relying on the published results in ref.^48^ and having performed the spectroscopic measurements at room temperature, we have considered a halved electrical conductivity *σ* = 2 × 10^7^ S/m for the 100 nm-thick metallizations in the full-wave simulations. However, this has produced only a slight reduction of the absorption level (considering an almost lossless polyimide dielectric layer) and a slight broadening of the bandwidth (see Supplementary Fig. S7).

Instead, the dielectric losses of the polyimide spacer have been found to have a pronounced effect on the simulated results; indeed, the permittivity and loss tangent selected in the simulations were based on the datasheet reported in ref.^31^. In the literature, there are many published papers^33–38^ in which the polyimide is treated as a dielectric with an a priori assumed permittivity value at THz frequencies. In the light of the foregoing considerations, we have determined a different value for the loss tangent of the polyimide, through parametric sweeps in order to match the measurements. We have found an estimated loss tangent *tan δ* = 0.086 to which corresponds a complex relative permittivity *ε*_*r*_ = 2.9 − 0.25 *j* for the polyimide layer. Figure 3(b) shows the comparison between the simulations with the modified permittivity and the experiments: a better agreement is evident, with a remarkable resonance at around 1.092 THz.

## Discussion

We have simulated and microfabricated an ultra-thin narrow-band MMA in the THz regime: its performance has been experimentally characterized by using a custom collimated configuration reflection-mode THz-TDS.

The realization of the MMA needs five main fabrication steps with only one lithographic phase: because the bottom layer is a continuous metal film, no alignment between the metal layers is needed. Therefore, the fabrication process is noticeably simplified with respect to other available solutions, for example that presented in ref.^23^ which implies the control of misalignment errors between the two stacked crossed-shaped metallizations, resulting in lower production time and costs. In addition, the lower ground plane avoids the influence of the support Si substrate that produced in ref.^23^ uncertainty in the definition of the unit cell, as well as a non-negligible asymmetry in the propagation direction due to the bianisotropy of the ERR design.

In order to experimentally characterize the MMA, we have modified a commercial Menlo Systems TERA K15 standard transmission configuration by inserting a fixed angle aluminum reflector in the collimated THz beam path. This permits the collimation of THz radiation on the sample/reference with a specific incidence angle, allowing investigating the MMA under oblique incidence. The devised reflection-mode THz-TDS set-up is polarization-maintaining because it does not use parabolic mirrors to manipulate the THz beam, allowing the characterization of polarization-sensitive devices and ensuring a wide reproducibility of the measurements.

The experimental results, carried out as a function of the azimuthal angle, have proved the high polarization insensitivity of the MMA even under 16° oblique incidence with absorbance levels comprised between 67% and 74% at 1.092 THz and a full-width-at-half-maximum (FWHM) of the absorption band of about 0.2 THz. The observed mismatch of the experimental FWHM with the full-wave simulations has been found to chiefly depend on the value of dielectric losses of the polyimide spacer. A good agreement with the experimental results has then been obtained by adjusting the value of the loss tangent of the polyimide spacer to tan *δ* = 0.086.

Furthermore, the proposed ultra-thin MMA has been realized with a highly flexible polyimide middle layer which expands its uses in non-planar applications. The MMA can be made actually flexible by spin-coating a further layer of polyimide on the Si substrate^39,40^, which represents only a mechanical support, not actively contributing to the absorption mechanism. The MMA can thus be regarded as a flexible metasurface which can be wrapped around non-planar surfaces and exploited to cover objects of considerable size.

These features make the device particularly suited for spectroscopic applications in several fields such as detection of explosive materials, THz imaging, electromagnetic cloaking, medical diagnoses, and spectrally selective security detection. The ultra-thin profile, the narrow spectral band of absorption and the polarization insensitivity, as well as the potential flexibility, open up also interesting prospects in the design of wearable THz MMAs.

## Methods

### Numerical simulation

The proposed MMA was simulated by applying the finite-element method (FEM), using the RF module of the commercial software package COMSOL Multiphysics. To this end, a single unit cell has been considered by applying Floquet periodic boundary conditions for the side boundaries perpendicular to the plane of the MMA to model a periodic structure. Perfectly matched layers (PMLs) are employed on the top and bottom of the unit cell to absorb the excited mode from the source port and any higher-order modes generated by the periodic MMA. Port boundary conditions are applied on the interior boundaries of the PMLs. For the excitation at the source port, an obliquely incident plane wave illumination with transverse electric polarization (i.e., the electric field is parallel to the MMA interface) and 1 W input power, constant over the THz band of interest, were used.

### Fabricated prototype

**Figure 4:**
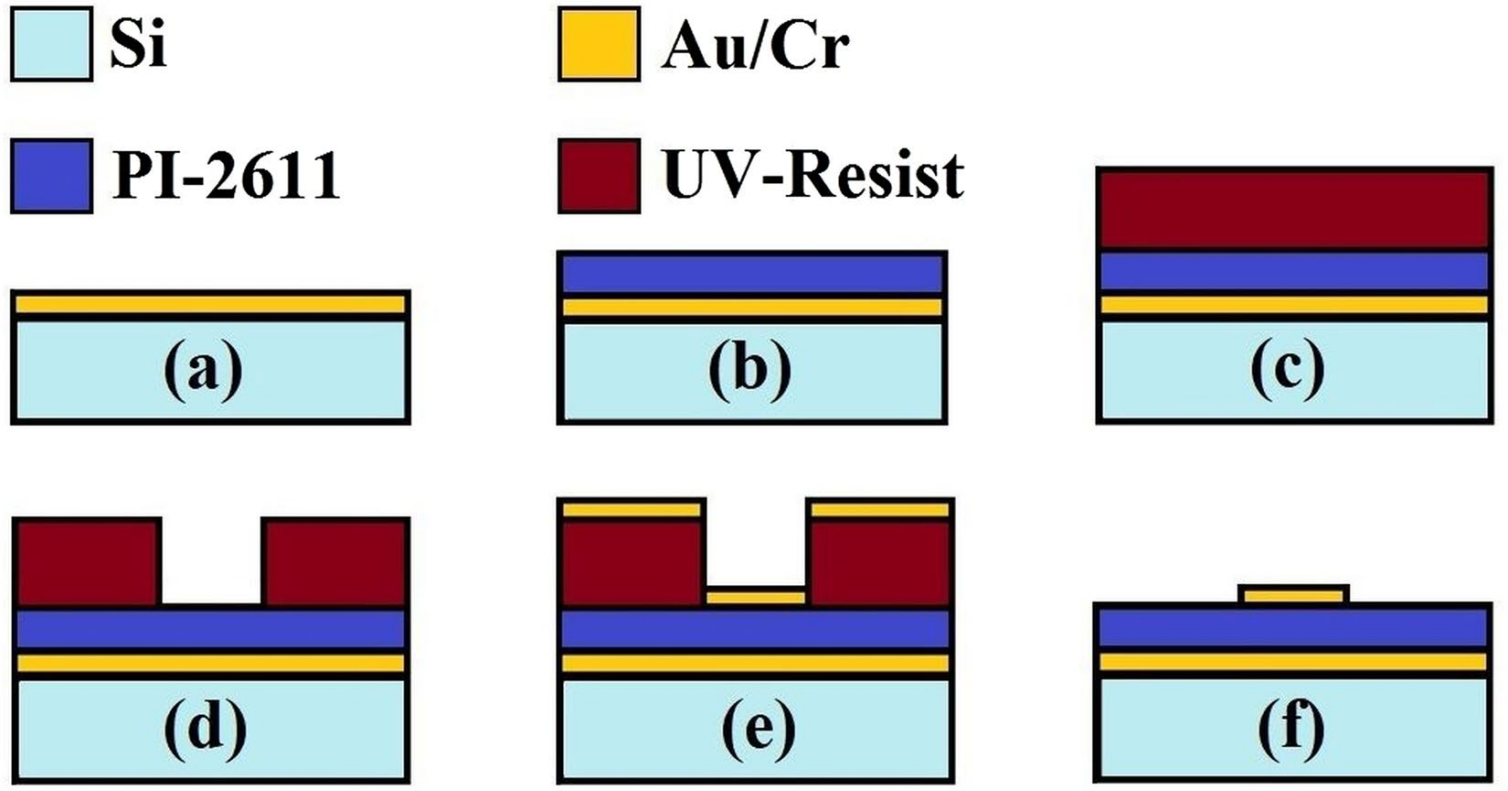
Schematic cross-sectional view of the fabrication process flow of a single unit cell. Steps of the fabrication process flow: (**a**) thermal evaporation of metal Au/Cr; (**b**) deposition of polyimide PI-2611; (**c**) deposition of UV-resist by spin-coating; (**d**) UV patterning process; (**e**) second thermal evaporation of metal Au/Cr; (**f**) removing of residual UV-resist by acetone.

In order to manufacture the device, we adopted the top-down fabrication approach using standard microfabrication techniques. The MMA sample was fabricated on a high-resistivity 360 *μ*m-thick silicon substrate that also serves as a support for the structure. The manufacturing procedure consisted of five main fabrication steps: first (Fig. 4(a)), on the silicon substrate cleaned with a buffered HF solution, a metal film was deposited by gun evaporator (10 nm of Cr adhesion layer and 100 nm of Au metal mirror); subsequently a 5.4 *μ*m-thick polyimide (PI-2611, HD MicroSystems) middle layer was deposited onto the Au film by spin-coating and cured in vacuum oven at 250 °C (Fig. 4(b)). Then (Fig. 4(c) and (d)), on the polyimide layer, the geometry of the unit cell, a matrix consisting of 6 cm × 6 cm of total active area (750 × 750 unit cells), was patterned with a UV-inverted resist (AZ 5214 E, MicroChemicals) using standard lithography process (Mask Aligner Karl Suss MA160); finally (Fig. 4(e)), a second metal layer was evaporated (10 nm Cr adhesion layer and 100 nm of Au) obtaining the final structure after lift-off procedure in acetone (Fig. 4(f)).

**Figure 5:**
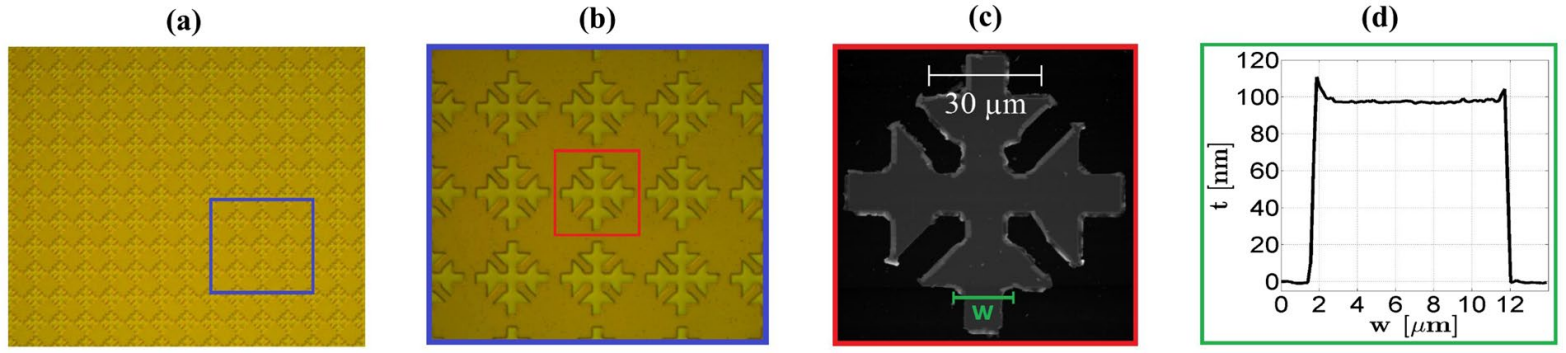
Optical microscope image illustrations and AFM inspection of the fabricated MMA. (**a**) Optical image of MMA large area; (**b**) optical image of ERRs details; (**c**) AFM inspection of a single unit cell; (**d**) AFM characterization of the arm width *w* = 11 *μ*m profile, where *t* = 100 nm is the metal thickness.

In Fig. 5(a) and (b), the optical microscope images of the completed device are reported, while in Fig. 5(c) and (d), the Atomic Force Microscopy (AFM) inspection of the unit cell shows that we reached a good control in the process fabrication flow obtaining a good definition of the tips and a sharp vertical wall of the final metal film.

### Data availability

The datasets generated during and/or analyzed during the current study are available from the corresponding authors on reasonable request.

## Electronic supplementary material

Supplementary Information
