# Supplementary material for: Polarization-maintaining reflection-mode THz time-domain spectroscopy of a polyimide based ultra-thin narrow-band metamaterial absorber
Source: Sci Rep. 2018 Jan 31;8:1985. doi: 10.1038/s41598-018-20429-7 (PMC5792614; doi:10.1038/s41598-018-20429-7)
Supplement: Supplementary file 1 — Supplementary Information [file 41598_2018_20429_MOESM1_ESM.pdf]

Supplementary Information

Polarization-maintaining reflection-mode THz time-domain spectroscopy of a polyimide based ultra-thin narrow-band metamaterial absorber

Maria Denise Astorino[1,*], Renato Fastampa[2,3], Fabrizio Frezza[1], Luca Maiolo[4], Marco Marrani[4], Mauro Missori[3,†], Marco Muzi[1,5], Nicola Tedeschi[1], and Andrea Veroli[1,6]

[1]Department of Information Engineering, Electronics and Telecommunications, “La Sapienza” University of Rome, Via Eudossiana 18, 00184 Roma, Italy
[2]Dipartimento di Fisica, “La Sapienza” University of Rome, Piazzale Aldo Moro 5, 00185 Roma, Italy
[3]Istituto dei Sistemi Complessi, Consiglio Nazionale delle Ricerche, Unità Sapienza, Piazzale Aldo Moro 5, 00185 Roma, Italy
[4]Istituto per la Microelettronica e Microsistemi - Consiglio Nazionale delle Ricerche, Via Fosso del Cavaliere 100, 00133 Roma, Italy
[5]Engineering Departmental Faculty, Campus Bio-Medico University of Rome, Via Alvaro del Portillo 21, 00128 Roma, Italy
[6]Centro Fermi - Museo Storico della Fisica e Centro Studi e Ricerche “Enrico Fermi”, Piazza del Viminale 1, 00184 Roma, Italy
[*]mariadenise.astorino@uniroma1.it
[†]mauro.missori@isc.cnr.it

S.1 Experimental absorbance spectra under 16° oblique incidence in TE polarization as a function of the azimuthal angle φ.

In order to demonstrate the reproducibility of the spectral data measured by means of the polarization-maintaining reflection-mode THz time-domain spectroscopy (THz-TDS) system, we reported the absorbance spectra obtained under 16° oblique incidence in transverse electric (TE) polarization for different azimuthal angles φ of 0, 22.5, 45, 67.5, and 90 degrees.

All spectroscopic measurements were performed at room temperature and at a relative humidity (RH) between 3.5% and 4.7%, to reduce the water vapor contribution particularly present in the frequency band of interest.

The Supplementary Figs. S1-S6 show the comparison between two different absorbance spectra $A(\omega)$ (see main text) measured at specific values of RH, temperature, and azimuthal angle, illustrating the reproducibility of the measurements. It should be noted that all the plots shown in the Supplementary Figs. S1-S6 represent the absorbance spectra obtained by applying the fast Fourier transform (FFT) to the acquired signals in the time-domain. The small differences that are present on the tails of the absorbance spectra are due to slight variations of the baseline. These do not significantly alter the relevant physical information that is contained in the position, amplitude, and shape of the absorbance peaks.

Bearing in mind that a THz-TDS set-up operating in reflection-mode has considerable criticality compared to the more easily configurable transmission-mode set-up [S1], we have reached a noticeable quality of the measurements. This is allowed by the adopted reflection-mode THz-TDS configuration, properly designed with the smallest number of mobile optical and mechanical parts (mirrors, lenses, and mechanical mountings), to avoid alignment errors. These might result in differences in THz pulses path length and phase errors, thus altering the measured signals and making difficult the comparison with the theoretical predictions.

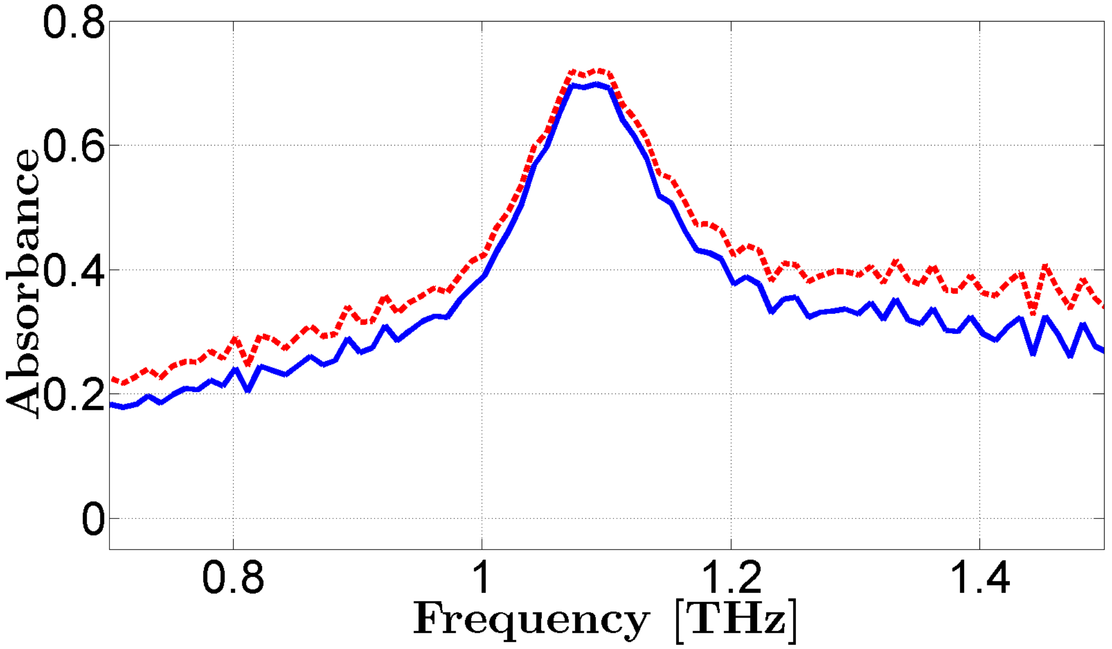

Supplementary Figure S1. Experimental absorbance spectra under 16° oblique incidence in TE polarization with azimuthal angle $\varphi = 0°$. The measurements have been performed at 23.7 °C and RH=4.7%.

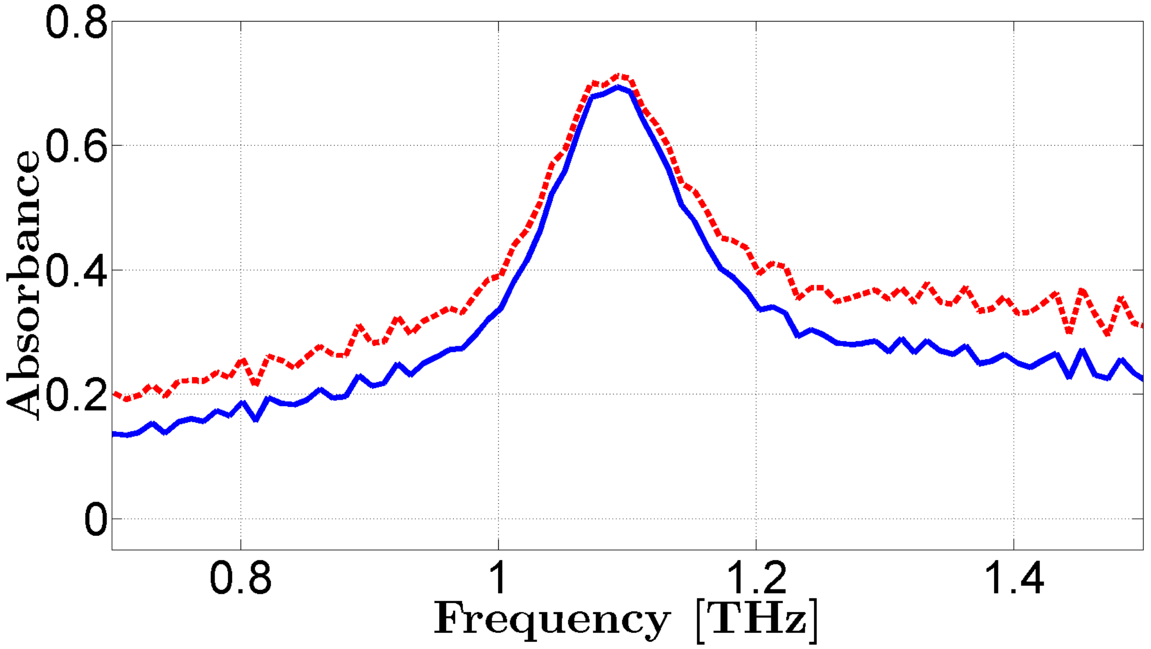

Supplementary Figure S2. Experimental absorbance spectra under 16° oblique incidence in TE polarization with azimuthal angle $\varphi = 0°$. The measurements have been performed at 23.8 °C and RH=3.5%.

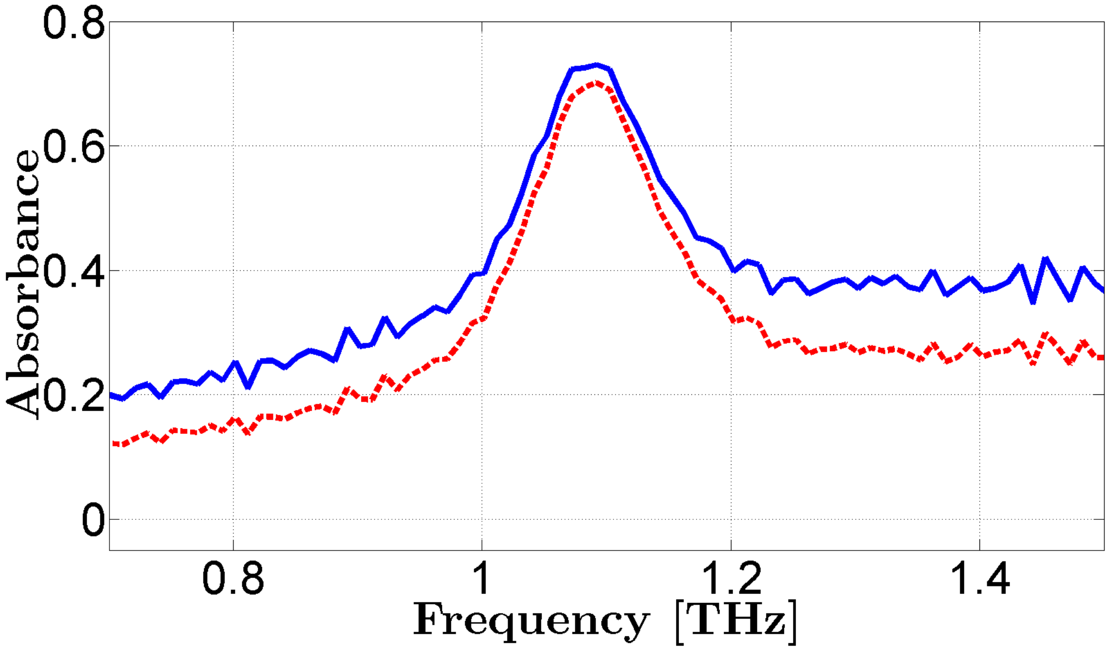

Supplementary Figure S3. Experimental absorbance spectra under 16° oblique incidence in TE polarization with azimuthal angle $\varphi = 22.5°$. The measurements have been performed at 23.6 °C and RH=4.2%.

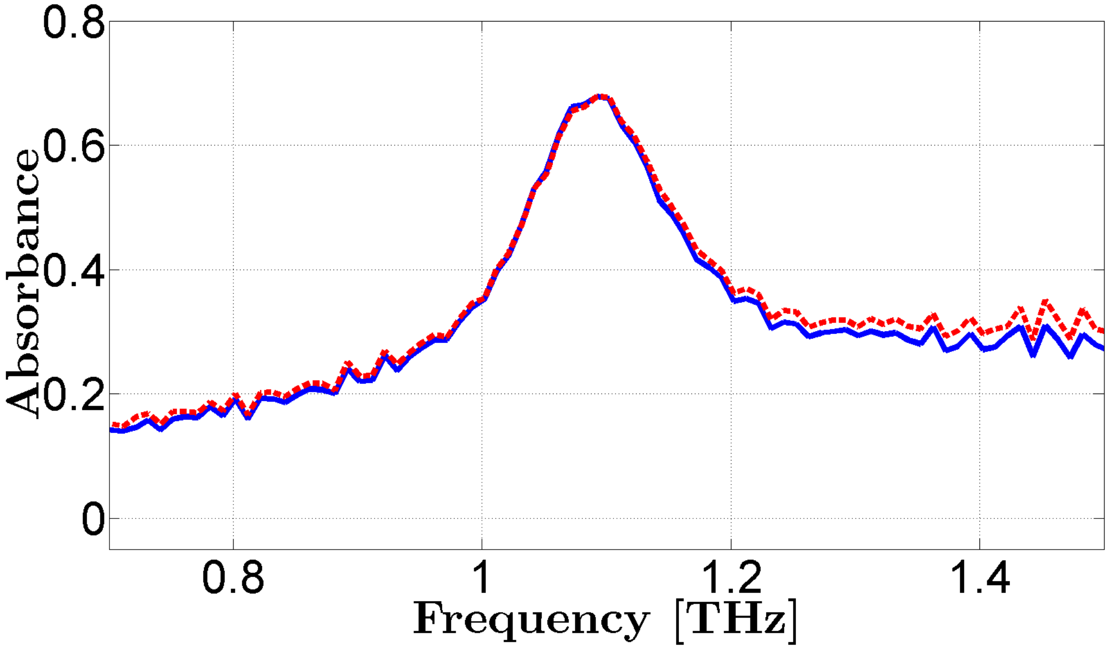

Supplementary Figure S4. Experimental absorbance spectra under 16° oblique incidence in TE polarization with azimuthal angle $\varphi = 45°$. The measurements have been performed at 23.7 °C and RH=4.0%.

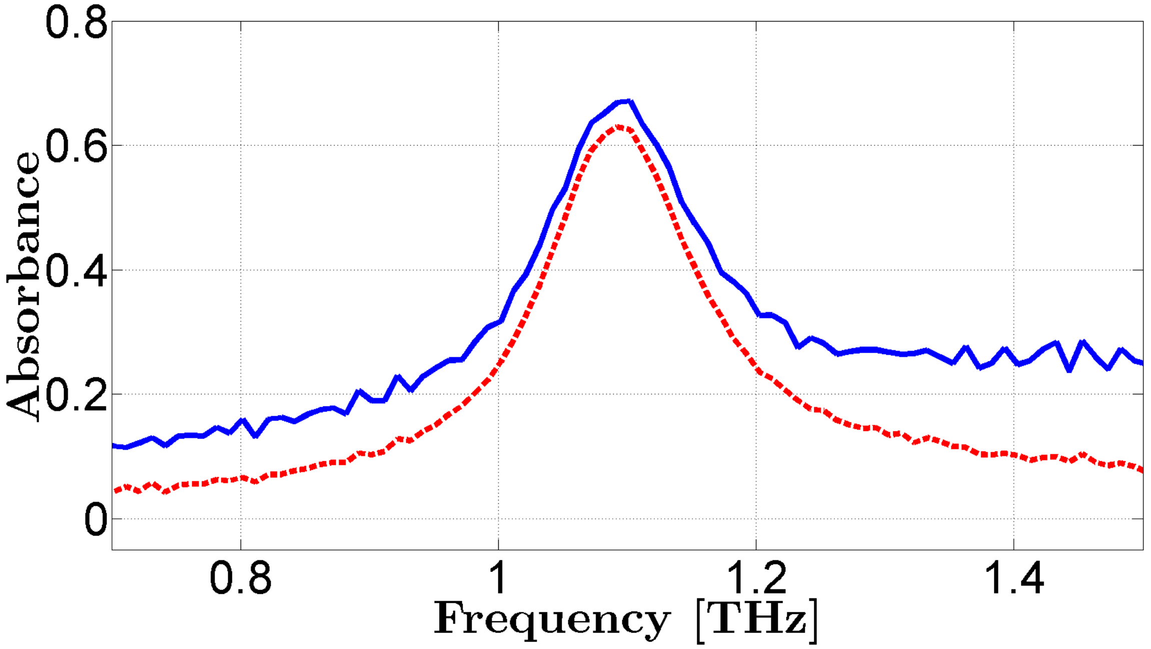

Supplementary Figure S5. Experimental absorbance spectra under 16° oblique incidence in TE polarization with azimuthal angle $\varphi = 67.5°$. The measurements have been performed at 23.7 °C and RH=3.8%.

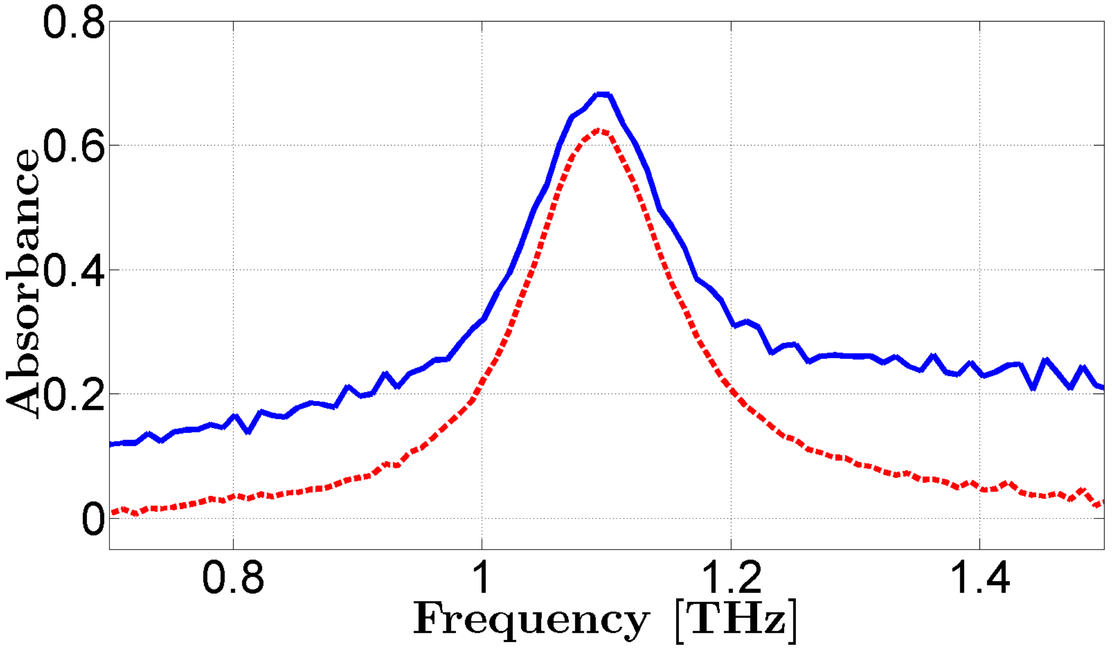

Supplementary Figure S6. Experimental absorbance spectra under 16° oblique incidence in TE polarization with azimuthal angle $\varphi = 90°$. The measurements have been performed at 23.8 °C and RH=3.6%.

S.2 Effect of higher Ohmic losses of the metal layers at THz frequencies.

In order to analyze the discrepancy between the measured and simulated absorbance spectra, we have considered the effect of lower conductivity of gold at THz frequencies. In particular, in Ref. S2, the conductivities of thin Al, Au, and Ag films measured via their transmission at THz frequencies, have been found much smaller than their bulk dc values, especially in the case of the thinner films and Al films. More specifically, the conductivity of 85 nm and 150 nm-thick Au films has been measured at 77 and 295 K. At the temperature of 295 K, the conductivity of 85 nm-thick Au film results of 1.5×10^7 S/m, while for a 150 nm-thick Au film is of 3.1×10^7 S/m. Since we performed the spectroscopic measurements at room temperature (see Supplementary Figs. S1-S6), in the full-wave simulations, we initially considered an electrical conductivity of 4.09×10^7 S/m for the 100 nm-thick metallizations, corresponding to the Au bulk conductivity dc value. We have then tested the effect of higher Ohmic losses on the absorbance behavior through parametric simulations as a function of the electrical conductivity σ.

In the Supplementary Fig. S7, it is highlighted how a halving of the electrical conductivity ($\sigma = 2 \times 10^7$ S/m) produces only a slight reduction of the absorption level and a slight broadening of the bandwidth when considering an almost lossless polyimide dielectric layer with a relative permittivity $\varepsilon_r = 2.9 - 0.0058j$ under 16° oblique incidence in TE polarization. Consequently, the dielectric losses of the polyimide spacer represent the main reason for the mismatch with the measurements.

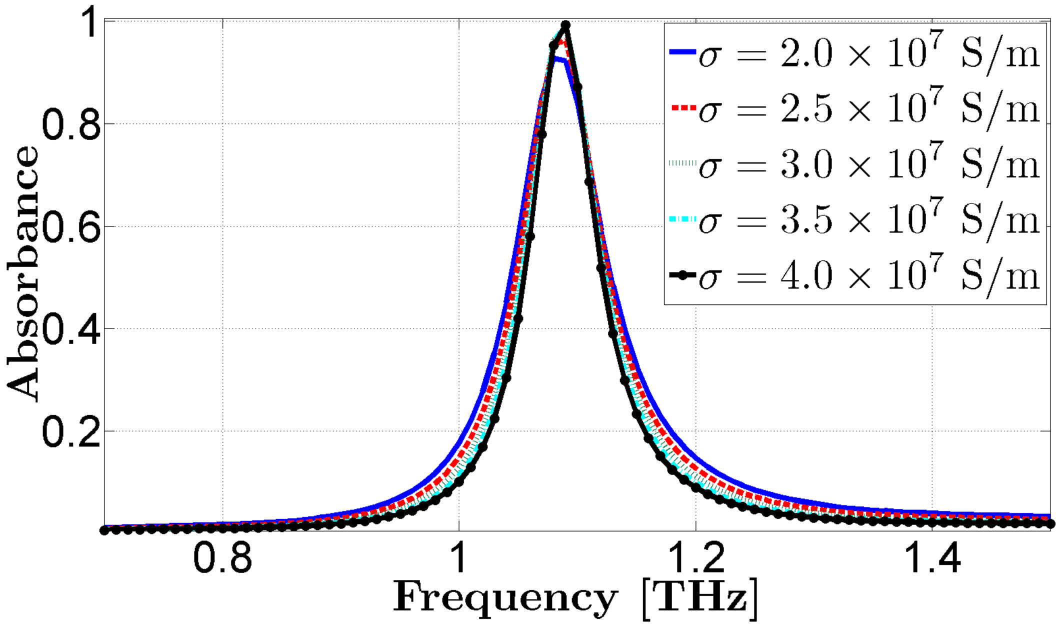

Supplementary Figure S7. Effect of higher Ohmic losses of the metal layers under 16° oblique incidence in TE polarization. Absorbance spectra as a function of the electrical conductivity σ of the 100 nm-thick metal layers, with a relative permittivity $\varepsilon_r = 2.9 - 0.0058j$ for the polyimide spacer.

References

S1. Jepsen, P. U., Cooke, D. G. & Koch, M. Terahertz spectroscopy and imaging–modern techniques and applications. *Laser & Photonics Rev.* **5**, 124–166 (2011).

S2. Laman, N. & Grischkowsky, D. Terahertz conductivity of thin metal films. *Appl. Phys. Lett.* **93**, 051105 (2008).
